# Effects of Morning Strength and Power Priming Exercises on Afternoon Physical and Cognitive Performance in Male Rugby Union Players

**DOI:** 10.1002/ejsc.70286

**Published:** 2026-11-10

**Authors:** Jamie Knight, Mark Russell, Daniel Cunningham, Christian Cook, Mark Waldron, Liam Kilduff

**Affiliations:** ^1^ Applied Sports Technology Exercise and Medicine Research Centre (A‐STEM) Faculty of Science and Engineering Swansea University Swansea UK; ^2^ Team Durham Durham University Durham UK; ^3^ Faculty of Social and Health Sciences Leeds Trinity University Leeds UK; ^4^ Department of Sports Sciences School of Social Sciences Reykjavík University Reykjavik Iceland; ^5^ FSI Laboratory Football Science Institute Granada Spain; ^6^ Welsh Institute of Performance Science (WIPS) Swansea University Swansea Swansea UK; ^7^ Biomedical Discipline School of Science and Technology University of New England Armidale New South Wales Australia; ^8^ Hamlyn Centre Imperial College London UK

**Keywords:** cognitive function, potentiation, resistance training, speed, team sports

## Abstract

The objective of this study was to assess the effect of morning strength and power priming exercises on afternoon physical and cognitive performance in male rugby union players. On two occasions in a randomised, counterbalanced design, 25 male rugby union players completed morning physical (countermovement jump, 40 m linear sprint) and cognitive (rapid visual information processing; spatial working memory; paired associates of learning) assessments. Control (passive rest) or intervention (barbell back squat, 3 × 3 repetitions at 85% of one repetition maximum and barbell squat jump, 5 × 3 repetitions at 40% one repetition maximum) trials were implemented 5.5 h before afternoon assessments. Differences between morning and afternoon assessments were found in sprinting performance following intervention for 30 m fly (−0.08 ± 0.13 s, *p =* 0.004, ES: 0.615) and 40 m (−0.12 ± 0.17 s, *p =* 0.001, ES: 0.711) relative to control (0.03 ± 0.07 s, *p* = 0.022, ES: −0.476; 0.01 ± 0.09 s, *p* = 0.740, ES: −0.065, 30 m fly and 40 m, respectively). Afternoon countermovement jump flight time improved following intervention compared with control (2% vs. 0%, *p =* 0.012, ES: −0.53). No changes were found for cognitive assessments (*p* > 0.05). Morning strength and power priming exercises combining heavy‐load traditional and light‐load ballistic exercises effectively enhanced vertical jump and sprint performance, but not cognitive performance, in the afternoon in male rugby union players.

## Highlights

- The effect of morning exercise involving both heavy‐load and ballistic resistance training on afternoon physical and cognitive performance in male rugby union players has not been extensively examined to date.
- Implementing morning resistance exercises 5.5 hours prior to testing significantly enhances afternoon 40 m sprint and countermovement jump performance.
- While physical performance improved, morning resistance exercise did not significantly alter afternoon indices of attention or working memory.

## 1 Introduction

Match outcomes in rugby union (RU) can be influenced by several key performance indicators, incorporating both physical and technical‐tactical components (Cunningham et al. 2018). Effective ball carrying, for example, has been associated with successful match outcome and is partially underpinned by physical qualities, such as lower body peak power output and acceleration momentum (Cunningham et al. 2018; Waldron et al. 2013). Additionally, rugby players are required to adjust their actions in response to evolving attacking and defencive scenarios involving both teammates and opponents. These decisions must often be made within very short time frames and while experiencing considerable physical fatigue, highlighting the cognitive demands of the sport (Davidow et al. 2023). To mitigate this fatigue and exploit tactical opportunities, a window of opportunity may exist on competition day to acutely enhance physical performance, with morning exercise priming implemented five to 8 hours prior to competition being efficacious for improving physical performance in the afternoon (Cook et al. 2014; Russell et al. 2016; Harrison et al. 2019; Mason et al. 2020; González‐García et al. 2022; Harrison et al. 2021).

Among the various cognitive demands of the sport, attention and working memory (WM) are two important cognitive abilities in competitive rugby (MacDonald et al. 2016). Working memory refers to the limited capacity cognitive system responsible for temporarily holding and manipulating information while controlling attention and resisting distraction (Furley and Memmert 2012). Previous researchers have demonstrated that athletes with a high WM capacity are better able at focusing their attention on tactical decision‐making while blocking out irrelevant auditory distractions (Furley and Memmert 2012). Similarly, Scharfen and Memmert (Scharfen and Memmert 2019) reported WM capacity to be positively related to sport‐specific skills within elite youth footballers. Indeed, resistance exercise has been shown to acutely enhance both attention and WM (Huang et al. 2022; Tsuk et al. 2019), although the effects of morning exercise priming on these specific cognitive domains remain unclear. To date, morning priming studies have typically evaluated broader cognitive domains rather than isolated WM capacity. For instance, professional cricketers displayed improved Stroop test performance (−3.83 s, 7.4%), a measure of selective attention following morning resistance exercise priming (Nutt et al. 2022). Conversely (Russell et al. 2016) reported no significant improvements in a different cognitive quality and processing speed, when measuring reaction time following morning exercise priming in male RU players. Importantly, because tasks, such as the Stroop test and simple reaction time metrics, capture distinct, overarching aspects of cognitive performance, there is a need for more sensitive, standardized assessment batteries, such as the Cambridge Neuropsychological Test Automated Battery (CANTAB), to precisely isolate working memory and attention. Consequently, the effects of morning resistance exercise priming on these targeted, established WM tasks have not yet been investigated.

From a neuromuscular perspective, improvements in countermovement‐jump (CMJ) peak power (+2.7%) and 40 m sprints (+1.3%) have been reported 6 hours after morning heavy resistance priming (Cook et al. 2014). More recently, morning lower body resistance priming (i.e., trap bar deadlifts, 6 × 4 repetitions up to 85% repetition maximum; RM) enhanced afternoon sprinting performance (−0.07 s, 1.2%) and jump height (+1.1 cm, 2.5%) within professional male cricketers (Nutt et al. 2022). Similarly, (Loturco et al. 2025) demonstrated that elite female rugby players exhibited enhance CMJ height performance, faster 40 m sprint times, and improved change of direction performance following morning resistance priming. Several mechanisms, such as increased mechanical stiffness, motor neuron activation, and increased fibre sensitivity to calcium ions, have been proposed to explain the efficacy of resistance exercise priming for enhanced afternoon performance (Harrison et al. 2019). Likewise, diurnal changes in hormonal status have been suggested to play a substantial role (Cook et al. 2014).

Low‐volume, and high‐load (≥ 85% 1RM) traditional exercise or low‐load (30%–40% 1RM) ballistic exercise have been recommended as most effective in a recent review (Harrison et al. 2019; Pereira et al. 2025). However, the potential interaction of time and intervention warrants consideration also, as heavy loads have been shown to be beneficial within some (Cook et al. 2014; González‐García et al. 2022 Harrison et al. 2021; Nutt et al. 2022), but not all time periods (Russell et al. 2016; Stavropoulos et al. 2025). Additionally, it has been proposed that movement‐specific activity be performed within priming sessions as these appear to be more effective in comparison to general, non‐specific activity (Mason et al. 2020). A notion supported by previous performance improvements in jumping, throwing, and sprinting when the priming activity implemented a similar movement pattern (Cook et al. 2014; Villarreal et al. 2007; Mason et al. 2017). Contrary to this, (Russell et al. 2016) reported that upper‐body priming improved subsequent lower‐body repeated sprint performance in professional rugby players, suggesting non‐specific strategies may also offer ergogenic benefits. However, because rugby union involves a diverse range of movement demands, the true efficacy of movement specific versus general priming remains unclear. To address this ambiguity and maximise the potential for acute transfer to lower limb explosive metrics, the present study utilised a combined heavy and ballistic lower body priming protocol to directly match the movement patterns of the afternoon physical performance assessments.

The potential for morning resistance exercise priming to acutely improve indices of cognitive performance may be particularly beneficial for RU players on game day. Therefore, this study aimed to investigate the effect of a morning strength and power priming exercises on afternoon cognitive and physical performance in male RU players. We hypothesised that a morning strength and power priming exercises would influence afternoon physical and cognitive performance.

## 2 Materials and Methods

Following institutional ethical approval, 25 amateur male rugby union players (age 21 ± 1 years, body mass 99.7 ± 12.5 kg, stature 1.86 ± 0.07 m) volunteered to participate in the current study. All participants competed in BUCS Super Rugby, with over 15 participants also involved in professional club academies and having represented their countries at international age‐grade level. Prior to participating, all individuals gave written informed consent and confirmed that they were healthy and free from injury. Participants were instructed that they could withdraw from the study at any point.

Each participant completed two separate assessment sessions under control (CON) and intervention (INT) conditions, each separated by 4 days in a randomised, counterbalanced, crossover design. Participants were instructed to keep their nutritional habits standardised and to avoid caffeine, alcohol, strenuous exercise and cognitively fatiguing tasks for 24 hours prior to every assessment session. To mitigate the effects of diurnal variation in performance, all experimental trials were administered at identical times of day (Figure 1) (Teo et al. 2011). Participants were familiarised with the assessment battery 7 days prior to the study commencing to ensure technical competency and procedural consistency and had over 12 months of resistance training and maximal sprint training experience.

**FIGURE 1:**
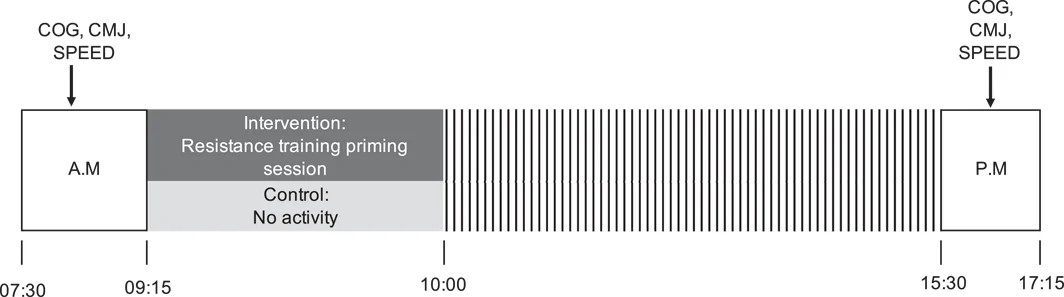
Overview of study protocol. 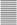 = passive recovery; A.M = morning base measures; CMJ = counter‐movement jump; COG = Paired associates of learning, spatial working memory, and rapid visual information processing assessments; P.M = afternoon measures; SPEED = 40 m linear sprint.

Following arrival at the testing venue, individuals undertook a sequence of three cognitive assessments via the Cambridge Neuropsychological Test Automated Battery (Cambridge Cognition Ltd) (CANTAB). These were undertaken on an iPad in a quiet room with minimal distractions and have been found to be both valid and reliable assessments of cognitive ability (Karlsen et al. 2022). The CANTAB test administration is fully automated, with participants being provided with on‐screen text instructions and voiceover guidance for each assessment, explaining response requirements and task goals. The participants were sat at a comfortable distance from the iPad and were instructed to use the index finger of their dominant hand during the tests. Following these assessments, participants completed a standardised dynamic warm‐up consisting of active mobility exercises (dynamic leg swings, linear and lateral lunges, glute bridges and dynamic calf stretching) and a 5‐min moderate cycle followed by the countermovement jump (CMJ) assessment. Participants then completed a standardised sprint warm‐up consisting of progressive jogging and running drills (pogo jumps, A‐skips, B‐skips, scissor bounds) and three progressive 40 m efforts at 70%, 80% and 90% of perceived maximal effort. Following this, the 40 m linear sprint assessment was completed on an artificial grass pitch. The following trial allocation, participants performed either the intervention priming (INT) or underwent passive recovery (CON). A standardised 5.5‐h rest interval was observed across both arms prior to participants completing the afternoon evaluation battery in the same sequence as the morning assessment session.

For INT, participants completed lower‐body resistance exercise (Table 1) following a standardised warm‐up (∼8 min) that required completion of dynamic stretching and progressive cycling. Loads for the intervention were calculated from a recent (< 2 weeks prior) 1RM back squat assessment. All 1RM testing was performed according to UKSCA standards, requiring participants to squat until the femur reached parallel with the floor (i.e., hips and knees flexed to parallel depth). This depth was standardised across all participants and verified by an accredited strength and conditioning coach. During the priming intervention, this depth was also the target coached to all participants, and verbal feedback was continuously provided to ensure this standard was strictly maintained throughout all intervention sets. Participants were instructed to move the bar as fast as possible during the concentric phase of each repetition, while maintaining proper technique throughout.

**TABLE 1:** Priming intervention completed by participants.

Exercise	Sets	Reps	Intensity (% of 1 RM)	Rest (minutes)
Barbell back squat	1	3	50	1
	1	3	75	3
	3	3	85	3
Barbell squat jump	5	3	40	3

Abbreviation: RM = repetition maximum.

### 2.1 Cognitive Performance Assessment

Extensively validated across diverse clinical cohorts, CANTAB measures show established test‐retest reliability when benchmarked against classic neurocognitive instruments (Karlsen et al. 2022; Gonçalves et al. 2018; Gonçalves et al. 2016; Smith et al. 2013; Torgersen et al. 2012). The CANTAB testing battery remained standardised throughout all assessment sessions, administered in an identical sequence consisting of Paired Associates Learning (PAL), followed by Rapid Visual Information Processing (RVP) and concluding with Spatial Working Memory (SWM). Table 2 summarises all cognitive assessment outcomes along with their corresponding definitions.

**TABLE 2:** Cambridge cognition cognitive assessment outcomes and definitions.

Test outcome	Outcome abbreviation	Definition
Paired associate learning total errors adjusted	PALTEA28	No. of times the subject chose the incorrect box for a stimulus on assessment problems, plus an adjustment for the estimated no. of errors they would have made on any problems, attempts and recalls they did not reach
Paired associate learning, first attempt memory score	PALFAMS28	No. of times a subject chose the correct box on their first attempt when recalling the pattern locations.
Paired associate learning, mean errors to success	PALMETS28	The mean no. of attempts made by a subject needed for them, to successfully complete the stage.
Rapid visual information processing response sensitivity	RVPA’	A’ prime is the signal detection measure of a subject's sensitivity to the target sequence, regardless of response tendency.
Rapid visual information processing median response latency	RVPML	The mean response latency of a correct response to a target sequence.
Spatial working memory strategy	SWMSX	The no. of times a subject begins a new search pattern from the same box they started with previously. Insight into search strategy used by the subject.
Spatial working memory total errors, 4‐, 6‐, and 8‐item	SWMTE468	Total errors: The total no. of times a box is selected that is certain not to contain a token and therefore should not have been visited by the subject for the 4, 6 and 8 token trials.
Spatial working memory total errors, 12‐item	SWMTE12	Total errors: The total no. of times a box is selected that is certain not to contain a token and therefore should not have been visited by the subject for the trials with only 12 tokens.
Spatial working memory between errors, 4‐, 6‐, and 8‐item	SWMBE468	Between errors: The no. of times the subject incorrectly revisits a box which a token has previously been found. Calculated across all 4, 6 and 8 token trials.
Spatial working memory between errors, 12‐item	SWMBE12	Between errors: The no. of times the subject incorrectly revisits a box which a token has previously been found. Calculated across trials with 12 tokens only.

#### 2.1.1 Paired Associates Learning

Paired Associates Learning (PAL) evaluated visual memory, episodic recall, and new learning capabilities (Backx et al. 2020). Spanning approximately 8‐min, the task presents boxes sequentially in a randomised layout to uncover hidden visual patterns, which are subsequently shown centrally one by one. Subjects must select the initial location corresponding to each pattern, with corrective re‐presentations occurring automatically following mistakes.

#### 2.1.2 Rapid Visual Information Processing

Sustained attention was evaluated using the RVP paradigm (Backx et al. 2020). Throughout this 7‐min test, digits present sequentially at a pace of 100 digits per minute, prompting participants to watch for predefined target sequences and register a response whenever the concluding digit appears.

#### 2.1.3 Spatial Working Memory

The SWM paradigm evaluates working memory and executive control functions (Backx et al. 2020). Spanning approximately 4 minutes, the task initiated with coloured boxes displayed on screen, requiring participants to use a systematic process of elimination to uncover a single ‘token’ hidden within each box and collect them in a side column. Subjects are informed that tokens do not repeat within the same box colour, meaning previously successful boxes should not be revisited. Box colours and locations vary across trials to prevent the reliance on fixed search patterns.

### 2.2 Physical Performance Assessment

Evaluations of physical performance encompassed a 40‐m linear sprint alongside a countermovement jump (CMJ). Jump performance was captured using a Kistler force plate (model 9260AA, Kistler, Germany) adhering to established testing protocols (Owen et al. 2014). Jump performance was analysed using a custom‐built software program developed by Swansea University that adheres to the criteria previously determined by (Owen et al. 2014) for CMJ analysis. Countermovement jump height was quantified using flight time, which has been shown to be a valid and reliable indicator of jump performance (Gallardo‐Fuentes et al. 2016). Participants began each jump standing motionless with their hands on their hips, executing a self‐paced countermovement dip followed by an explosive push to maximise jump height. Participants were instructed and verbally encouraged to jump as high as possible on each attempt, with all jumps performed under standardised conditions with consistent instructions to minimise variability in jump strategy. A total of three jump repetitions were performed, recording the peak flight time for analytical purposes. For the linear speed assessment, a 40‐m test was administered on an artificial grass training surface using the participant's habitual footwear. Sprints began 0.5 m back from the starting plane following a verbal prompt, and performance times for 10 m, 30 m fly and 40 m were logged using electronic timing systems (Brower Timing Systems, USA). The fastest performance out of three valid trials was selected for the final dataset.

### 2.3 Statistical Analysis

All statistical evaluations were conducted using SPSS Statistics software (IBM Inc., USA, version 29), establishing statistical significance at *p* ≤ 0.05. Normality of the data was assessed using the Shapiro–Wilk test. Data following a normal distribution are presented as Mean ± Standard Deviation (SD). For variables where normality assumptions were violated (30 M Fly INT P.M and 40 M CON A.M), data are presented as Median (Interquartile Range [IQR]), and non‐parametric Wilcoxon Signed‐Rank tests were employed for analysis. Data sphericity was checked using Mauchly's test, applying Greenhouse–Geisser corrections if violations were detected. Two‐way repeated‐measures ANOVAs tested for condition‐by‐time (A.M vs. P.M) interactions. Confirmed interactions indicated that experimental trials modified responses, triggering simple main effect analyses. Bonferroni‐adjusted post hoc comparisons and partial eta‐squared (*η* ^2^) values quantified significant outcomes. Paired *t*‐tests identified exact variations between temporal markers and conditions upon observing significant interactions, with Hedge's g effect sizes (ES) computed and interpreted as trivial (0.00–0.19), small (0.20–0.49), moderate (0.50–0.79), or large (≥ 0.80) (Cohen 1992).

## 3 Results

### 3.1 Sprint Performance

Figure 2A–C present the sprint data as a function of trial. Due to the presence of non‐normal distribution in specific subsets (30 M fly INT P.M and 40 M CON A.M), these data are reported as Median [IQR], and non‐parametric Wilcoxon signed‐rank tests were used for post hoc comparisons to ensure the validity of the 2‐way repeated measures ANOVA findings.

**FIGURE 2:**
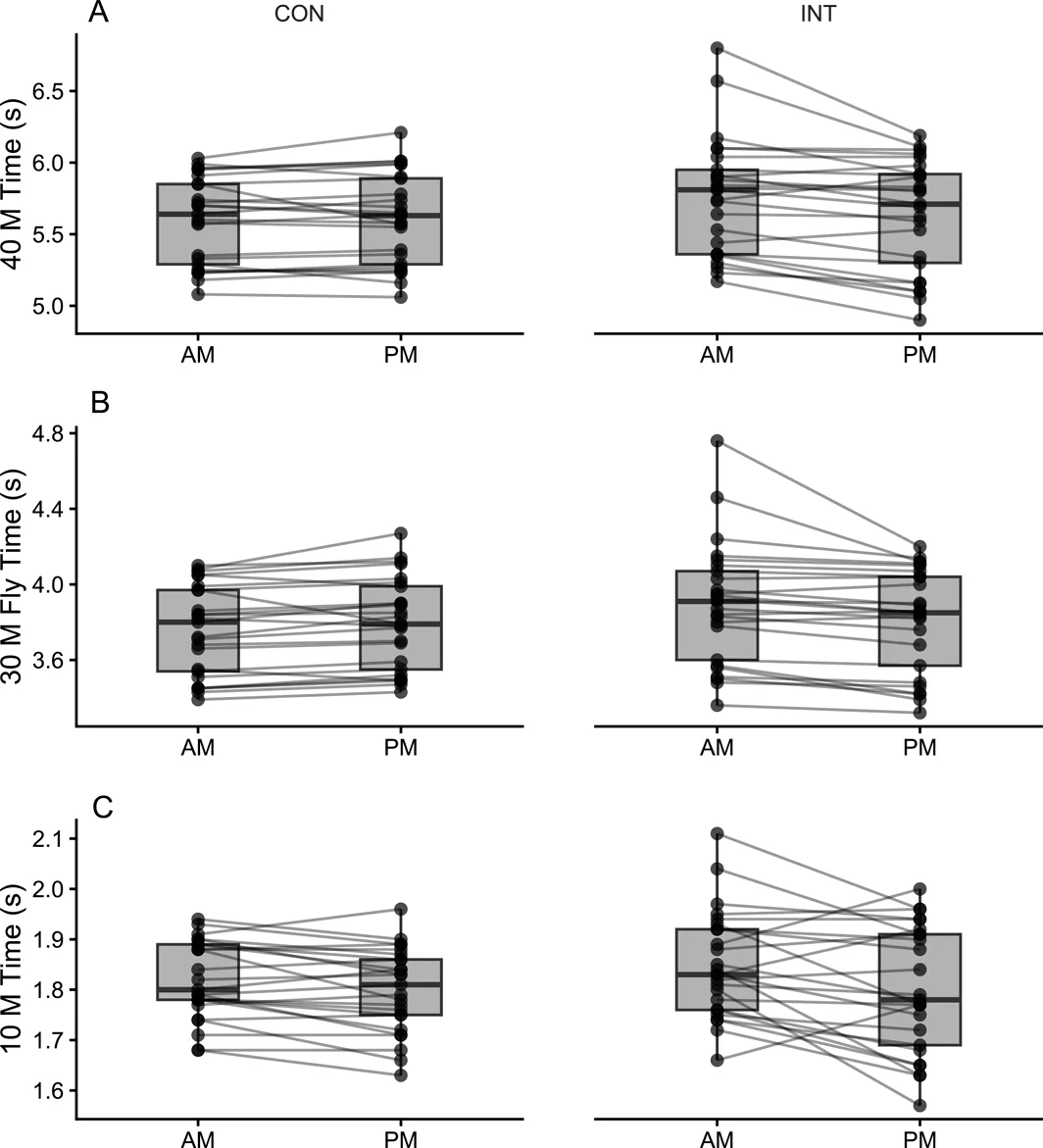
Sprint performance metrics across conditions and time: (A) 40 M, (B) 30 M Fly, and (C) 10 M. Data are presented as Box and Whisker plots showing the median, interquartile range (IQR), and full range. While 10 M data followed a normal distribution, Box and Whisker plots were utilised across all panels to provide a consistent and transparent representation of data distribution and to account for the non‐normality observed in the 30 M Fly and 40 M sprint subsets.

A significant interaction effect was found for both 30 m fly *(p* < 0.001, partial‐eta^2^ = 0.386) and 40 m sprints (*p* = 0.004, partial‐eta^2^ = 0.302). Post hoc analysis for A.M to P.M comparisons elicited superior performance improvements in 30 m fly following INT (Median change: −0.04 s [‒0.09, −0.03], *p* = < 0.001, ES: 0.615, moderate) compared to CON (Median change: 0.04 s [0.02, 0.06], *p* = 0.022, ES: −0.476). Similarly, for the 40 M sprint, INT showed significant improvements (Median change: −0.08 s [‒0.2, −0.01], *p* = 0.001, ES: 0.711, moderate), whereas no significant change was observed in CON (Median change: 0.03 s [‒0.03, 0.06], *p* = 0.332, ES: −0.065).

In addition, Table A in Supporting Information S1 presents the linear sprint data. It should be noted that baseline differences were evident for the 30 m fly and 40 m sprint split times between conditions. However, these were derived from the same 40 m sprint effort and may reflect between session variability rather than isolated differences in acceleration or maximal sprint performance.

### 3.2 Countermovement Jump Performance

A significant interaction effect was found for CMJ FT (*p* = 0.012, partial‐eta^2^ = 0.237) and was significantly influenced by time (*p* = 0.004, partial‐eta^2^ = 0.291). Similar responses were observed between trials at A.M (0.402 ms ± 0.561; *t*_(24)_ = 2.049, *p* = 0.052) and P.M (0.385 ms ± 0.053; *t*_(24)_ = −0.494, *p* = 0.626), but P.M data in INT exceeded CON (2% vs. 0%; *t*_(24)_ = −2.730, *p* = 0.012, ES: −0.53). Post hoc analysis discovered significant differences from A.M to P.M in INT (0.385 ms ± 0.053; *t*_(24)_ = −3.628, *p* = 0.001, ES: −0.7) compared to CON (0.405 ms ± 0.057; *t*_(24)_ = ‐0.10, *p* = 0.992) (Figure 3). Table B Supporting Information S1 presents the CMJ test data.

**FIGURE 3:**
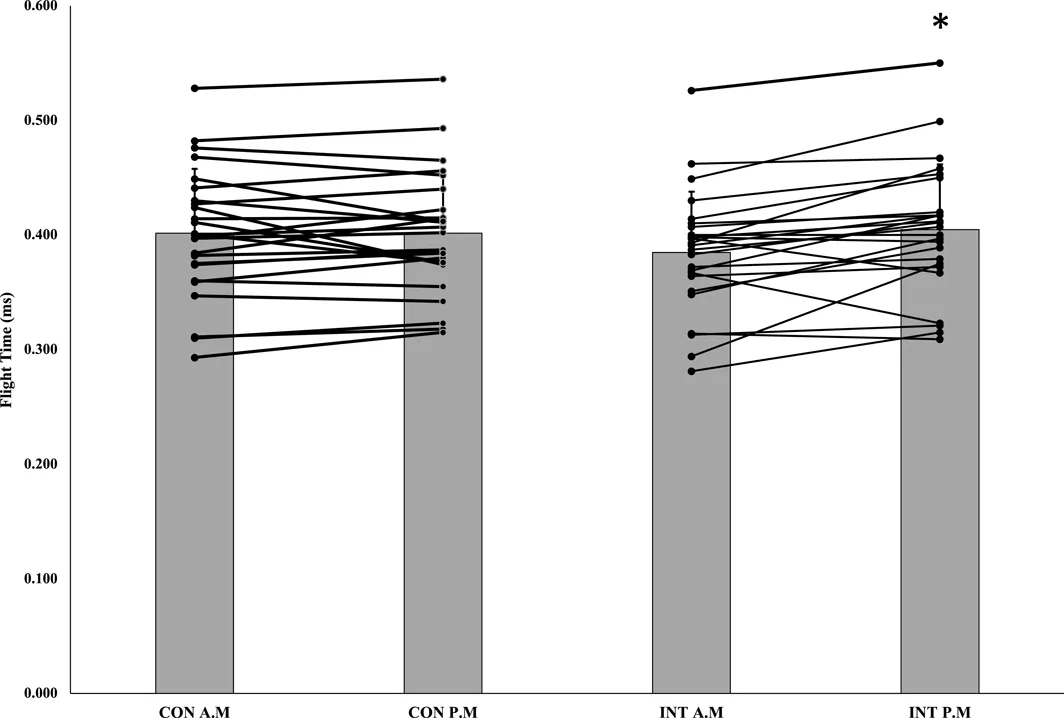
Mean ± SD CMJ flight time across conditions and time points. * = significant difference to A.M time point of trial condition; CON = Control; INT = Intervention.

### 3.3 Cognitive Assessments

No significant interaction effects were found for any indices of cognitive performance (PAL, RVP or SWM) (Table 3). Similar baseline values were observed across all cognitive measures (*p* > 0.05). A significant time effect was found for RVP mean response latency of a correct response to a target sequence (RVPML) (F_(1,24)_ = 5.710, *p =* 0.025, partial‐eta^2^ = 0.192). Although non‐significant, response latencies reduced from A.M to P.M in CON (453.53 ± 75.27 vs. 436. 07 ± 50.82, ES: 0.33%, −3.75%) and INT (468. 24 ± 83.55 vs. 442.81 ± 52.82, ES: 0.34%, −5.56%). No other significant time effects were found in the cognitive test outputs. In addition, Table C Supporting Information S1 presents the CANTAB test data.

**TABLE 3:** Mean ± SD results and *p* values for interaction effects in PAL, RVP and SWM cognitive assessment outputs.

Test	Control		Intervention		*p* value Interaction effect
	A.M	P.M	A.M	P.M	
PALTEA28	9 ± 7	7 ± 8	10 ± 9	8 ± 8	0.589
PALFAMS28	14 ± 3	16 ± 3	14 ± 4	15 ± 4	0.771
PALMETS28	2 ± 1	1 ± 1	2 ± 1	1 ± 1	0.519
RVPA’	0.93 ± 0.05	0.94 ± 0.04	0.93 ± 0.05	0.94 ± 0.03	0.748
RVPML	453.53 ± 75.27	436.07 ± 50.82	468.24 ± 83.55	442.81 ± 52.82	0.661
SWMSX	9 ± 5	8 ± 5	9 ± 5	10 ± 5	0.065
SWMTE468	4 ± 7	3 ± 6	5 ± 6	6 ± 8	0.391
SWMTE12	23 ± 16	19 ± 11	19 ± 14	19 ± 14	0.245
SWMBE468	4 ± 6	3 ± 6	5 ± 6	6 ± 8	0.381
SWMBE12	22 ± 14	18 ± 11	18 ± 14	19 ± 14	0.293

## 4 Discussion

This study investigated the effects of morning strength and power priming exercises on afternoon physical and cognitive performance in male RU players. The results demonstrated that afternoon physical performance was significantly improved the following morning strength and power priming. However, no significant improvements in cognitive performance were noticed despite RVPML being influenced by time. These results suggest that heavy‐load traditional and ballistic lower body exercises can be effectively combined within a single morning priming session to enhance acute afternoon explosive capacity in rugby union players. Although the study was conducted specifically in male RU players, the intervention and outcome measures used in the present design reflect performance qualities that are relevant across a broader range of athletes. Therefore, while the findings should be interpreted within the context of RU, they may also hold practical relevance for other team sport athletes who require expression of lower limb explosive abilities.

Relative to CON, completing a combination of lower body strength and ballistic exercise enhanced CMJ flight time (0.385 ms ± 0.053, 2%) and 30 m fly and 40 m sprinting performance (−0.08 ± 0.13 s, 8%, −0.12 ± 0.17 s, 12%, respectively). These improvements in afternoon physical performance extend recent recommendations that morning resistance priming of low volume, high‐intensity loads can improve neuromuscular performance 6 hours later (Harrison et al. 2019). It has been reported physical performance improvements following both high intensity resistance training (> 80% 1RM) and ballistic exercise performed with maximal intent at lower intensities (≤ 45% 1RM) (Holmberg et al. 2023). The experimental study in Olympic female rugby seven players demonstrated that both light‐ (40% 1RM) and heavy‐load (80% 1RM) jump squats performed six hours prior to testing improved CMJ height, sprint speed, and change of direction performance (Loturco et al. 2025). However, that study examined ballistic exercise in isolation and reported greater perceived exertion following heavy‐load priming. In contrast, the present study combined heavy strength and ballistic exercises within the same morning priming session and observed meaningful improvements in afternoon sprint and jump performance in male team sport athletes. These findings suggest that an integrated loading strategy, specifically within the lower body, may be effective for enhancing afternoon neuromuscular output. It would be useful for future research to investigate whether the effects extend to upper body performance, are sex specific and the effect on amateur and professional athletes.

Similarly, a narrative review on priming exercises concluded that heavy resistance and ballistic modalities can independently enhance subsequent speed and power performance when implemented 2–48 hours before competition (Pereira et al. 2025). The authors also highlighted substantial heterogeneity in protocols and called for more ecologically valid sport‐specific investigations. The present study contributes to this literature by applying a combined heavy‐ballistic priming approach within a realistic 5–6 hours competition day timeframe in male team sport athletes. Thereby addressing gaps identified within the review and providing applied evidence within a team‐sport setting. Furthermore, while much of the existing priming literature focuses strictly on senior elite or professional cohorts, the participants in the present study comprised competitive amateur and university‐academy players. The positive acute physical enhancements observed here demonstrate that the ergogenic benefits of morning priming are not exclusive to elite professional environments with extensive resources. Instead, these strategies represent an accessible, practical, and low‐fatigue method that can be effectively deployed across various competitive performance tiers to optimise the same day physical output.

In the case of sprinting, our results provide further insight into the effects of morning resistance priming on afternoon sprinting performance, where there is currently a scarcity of research findings. (Cook et al. 2014) reported a decrease in 40 m sprint times (−0.02–0.08 s) whereby subjects completed a three‐RM in the barbell back squat and bench press exercises. In contrast, the current study demonstrates that significant sprint improvements may be found with sub‐intensity, which may be more feasible on the day of competition. Additionally, our findings of improved sprint performance following a lower body resistance training session despite recent suggestions that the ergogenic effects of priming are movement specific (Mason et al. 2020). Sprinting itself has been researched as a method of priming and has demonstrated positive results (Mason et al. 2020). Despite its practicality due to no equipment required, athletes may not wish to sprint maximally on the morning of competition. The results of the current study extend the agreement of previous literature, demonstrating that morning resistance exercise priming can improve afternoon sprinting performance and may display a greater ergogenic effect than sprinting alone (Russell et al. 2016; Nutt et al. 2022). This enhancement likely reflects a cumulative effect, as our methodology required participants to complete maximal baseline sprints and jumps immediately before the intervention. Given that sprinting ability is a key determinant of performance in numerous team sports, these findings may be of interest to practitioners working beyond RU, particularly where low volume and low fatigue strategies are required within congested training or competition schedules.

The literature is currently unclear on the effects of morning exercise priming on cognitive performance, with previous literature investigating reaction time only (Russell et al. 2016; Nutt et al. 2022). In the current study no significant improvements in cognitive performance, specifically reaction time, sustained attention and working memory were found, supporting previous findings within elite rugby players (Russell et al. 2016). Although non‐significant, lower response latencies were found in the afternoon following INT compared with CON (−5.56% vs. −3.75%, respectively) in the RVP assessment in the current study. Despite differing assessments, previous evidence in professional cricketers has shown significant improvements in reaction time following morning resistance priming (Nutt et al. 2022). The mechanisms underlying these findings remain unclear. Neither (Nutt et al. 2022) nor the present study included hormonal assessments; therefore, any role of testosterone in acutely influencing cognitive performance remains speculative. Furthermore, the lack of ecological validity of the cognitive assessments used is a noteworthy limitation of the current study. Additionally, the cognitive assessment battery comprised a sequence of continuous tests spanning approximately 19 minutes without structured rest intervals between tasks. Although this design captured a comprehensive profile of cognitive function, the prolonged period of continuous demands may have induced cumulative mental fatigue, potentially influencing subsequent performance measures. To address this, subsequent investigations should incorporate structured rest intervals between subtests or counterbalance task order. Future research investigating sporting skill performance such as passing accuracy under time pressure within RU may provide a more ecologically valid insight into the effects of morning strength and power priming on afternoon cognitive performance. Additionally, it is currently unknown whether a sporting skill‐based cognitive primer completed in the morning may improve afternoon performance. In support of integrating more ecologically valid protocols, (Modric et al. 2023) demonstrated the efficacy of match‐day priming strategies incorporating complex movement and reactive elements in elite soccer. In the case of RU, this may involve the completion of a three versus two attack and defence drill that has various cognitive requirements such as decision making and attention.

The mechanisms behind our observed results cannot be fully determined from the present study; however, previous researchers have discovered that morning resistance priming can have positive effects offsetting the diurnal decline of testosterone throughout the day, leading to enhanced physical and potentially cognitive afternoon performance (Cook et al. 2014; Nutt et al. 2022). The physical improvements reported despite no cognitive improvement suggest that other mechanisms may play a significant role. For example, muscle and body temperature also demonstrate diurnal variations throughout the day and correlate with physical performance indices (Pullinger et al. 2014, 2018). Furthermore, it has been suggested that neuromuscular responses may play a key role in the priming response (Harrison et al. 2019), which may partially explain the performance enhancements observed herein. Although the present design mirrored previous research protocols (Donghi et al. 2020), the requirement for participants to complete maximal sprinting and jumping in the morning may represent a methodological limitation. Although this procedure was standardised across conditions, maximal morning exercise may have independently contributed to either neuromuscular potentiation or residual fatigue prior to afternoon assessments. Therefore, the extent to which the observed afternoon improvements can be attributed solely in the morning priming exercises should be interpreted with some caution. Future research should seek to isolate the effects of morning maximal assessment from priming interventions. Despite the standardisation of CMJ testing across sessions, the use of flight time as the primary outcome means that subtle changes in jump strategy cannot be fully excluded. Notably, despite asking subjects to maintain habitual nutritional habits during assessment days in the current study, the timing and macronutrient composition of food during the passive recovery phase may have had detrimental effects on their afternoon cognitive performance (Mahoney et al. 2005). Specifically, postprandial fatigue may be driven by high‐glycaemic carbohydrate fluctuations causing reactive hypoglycaemia and altered tryptophan‐to‐serotonin pathways (Benton 2002; Spring et al. 1982), or lipid‐induced cholecystokinin secretion that suppresses central alertness (Wells et al. 1997). Because explicit nutritional tracking was not employed in the current study, future research should strictly control or quantify post‐exercise intake to isolate mechanical fatigue from diet‐induced central lethargy. Although participants were instructed to abstain from caffeine for 24 hours prior to testing, future research exploring afternoon cognitive performance should consider stricter dietary standardisation, including chronic ergogenic aid habits and precise meal timing (Walczak et al. 2024; McLellan et al. 2016). Due to the current mixed findings, further research investigating how cognitive performance may be acutely enhanced to support athletes on competition day is warranted.

### 4.1 Conclusion

We investigated the effects of morning strength and power priming exercises on afternoon physical and cognitive performance. Our results demonstrate that afternoon physical performance can be improved after a 5.5‐h time interval that followed morning low volume, lower body strength and power exercise in male rugby union players. Although these findings should be interpreted within the context of male RU, the performance characteristics targeted by the intervention are relevant to a wider range of athletes. Therefore, the present study may offer broader practical insight for coaches and practitioners seeking to optimise same day performance through morning exercise priming. Specifically, practitioners may consider opportunities to augment explosive physical performance in the afternoon by prescribing a combined heavy strength and low‐load ballistic lower body exercise session in the morning, approximately 5.5 hours prior to competition. Future research should explore whether the present findings generalise to other team sports and to female athletes when similar priming protocols are implemented, as previous studies in women have utilised differing exercise approaches. Conversely, our results did not support significant improvements in afternoon cognitive performance.

### 4.2 Practical Implications

- Morning strength and power priming exercises consisting of heavy strength and ballistic exercise can improve indices of afternoon physical performance when commenced ∼5.5‐h after the priming stimulus. This strategy may therefore have practical applications not only for competition preparation, but also for enhancing the quality of later training sessions when appropriately scheduled.
- Practitioners working with male team‐sport athletes can utilise low‐volume, high‐intensity, ballistic lower‐body resistance training during morning schedules to enhance afternoon sprint and jump outputs on match days.
- While the broader body of evidence regarding the effects of morning resistance exercise on afternoon cognitive performance remains mixed, the present findings demonstrate that this specific type of heavy and ballistic neuromuscular stimulus does not acutely influence these cognitive abilities.

## Funding

There was no financial assistance with the project. This research did not receive any specific grant from funding agencies in the public, commercial, or not‐for‐profit sectors. Swansea University supported this research by covering the costs for the Cambridge Cognition Ltd. Cognitive assessments. Cambridge Cognition Ltd. Had no influence on the study design, execution, reporting and publishing of results.

## Ethics Statement

Swansea University ethics committee approved this research (3 2023 7344 6572) on 24/8/2023. Following obtaining this approval, a recruitment period was followed from 25/8/2023 to 1/9/2023 prior to data collection beginning. All participants were healthy, injury free and volunteered and provided written informed consent prior to involvement. Participants were informed that they could withdraw from the study at any point.

## Conflicts of Interest

Please see separate document uploaded under “Supplementary Material Not for Review” section for Disclosure Form.

## Supporting information


Supporting Information S1

## Data Availability

The data that supports the findings of this study are available in the supplementary material of this article.

## References

[ejsc70286-bib-0001] Backx, R. , C. Skirrow , P. Dente , J. H. Barnett , and F. K. Cormack . 2020. “Comparing Web‐Based and Lab‐Based Cognitive Assessment Using the Cambridge Neuropsychological Test Automated Battery: A Within‐Subjects Counterbalanced Study.” Journal of Medical Internet Research 22, no. 8: e16792. 10.2196/16792. PMC7435628 32749999

[ejsc70286-bib-0002] Benton, D. 2002. “Carbohydrate Ingestion, Blood Glucose and Mood.” Neuroscience and Biobehavioral Reviews 26, no. 3: 293–308. 10.1016/s0149-7634(02)00004-0. 12034132

[ejsc70286-bib-0003] Cohen, J. 1992. “Statistical Power Analysis.” Current Directions in Psychological Science 1, no. 3: 98–101. 10.1111/1467-8721.ep10768783.

[ejsc70286-bib-0004] Cook, C. J. , L. P. Kilduff , B. T. Crewther , M. Beaven , and D. J. West . 2014. “Morning Based Strength Training Improves Afternoon Physical Performance in Rugby Union Players.” Journal of Science and Medicine in Sport 17, no. 3: 317–321. 10.1016/j.jsams.2013.04.016. 23707139

[ejsc70286-bib-0005] Cunningham, D. J. , D. A. Shearer , S. Drawer , et al. 2018. “Relationships Between Physical Qualities and Key Performance Indicators During Match‐Play in Senior International Rugby Union Players.” PLoS One 13, no. 9: e0202811. 10.1371/journal.pone.0202811. PMC6135371 30208066

[ejsc70286-bib-0044] Davidow, D. , M. Smith , T. Ross , et al. 2023. “Mental Fatigue Impairs Tackling Technique in Amateur Rugby Union Players.” International Journal of Sports Physiology and Performance 18, no. 9: 960–967. 10.1123/ijspp.2023-0159. 37463669

[ejsc70286-bib-0006] Donghi, F. , E. Rampinini , A. Bosio , M. Fanchini , D. Carlomagno , and N. A. Maffiuletti . 2020. “Morning Priming Exercise Strategy to Enhance Afternoon Performance in Young Elite Soccer Players.” International Journal of Sports Physiology and Performance 16, no. 3: 1–8. 10.1123/ijspp.2020-0094. 33401241

[ejsc70286-bib-0007] Furley, P. A. , and D. Memmert . 2012. “Working Memory Capacity as Controlled Attention in Tactical Decision Making.” Journal of Sport & Exercise Psychology 34, no. 3: 322–344. 10.1123/jsep.34.3.322. 22691397

[ejsc70286-bib-0008] Gallardo‐Fuentes, F. , J. Gallardo‐Fuentes , R. Ramírez‐Campillo , et al. 2016. “Intersession and Intrasession Reliability and Validity of the My Jump App for Measuring Different Jump Actions in Trained Male and Female Athletes.” Journal of Strength and Conditioning Research 30, no. 7: 2049–2056. 10.1519/jsc.0000000000001304. 27328276

[ejsc70286-bib-0009] Gonçalves, M. M. , M. S. Pinho , and M. R. Simões . 2018. “Construct and Concurrent Validity of the Cambridge Neuropsychological Automated Tests in Portuguese Older Adults Without Neuropsychiatric Diagnoses and With Alzheimer’s Disease Dementia.” Aging, Neuropsychology, and Cognition 25, no. 2: 290–317. 10.1080/13825585.2017.1294651. 28632037

[ejsc70286-bib-0010] Gonçalves, M. M. , M. S. Pinho , and M. R. Simões . 2016. “Test–Retest Reliability Analysis of the Cambridge Neuropsychological Automated Tests for the Assessment of Dementia in Older People Living in Retirement Homes.” Applied Neuropsychology:Adult 23, no. 4: 251–263. 10.1080/23279095.2015.1053889. 26574661

[ejsc70286-bib-0011] González‐García, J. , C. Latella , M. Aguilar‐Navarro , and B. Romero‐Moraleda . 2022. “Effects of Resistance Priming Exercise on Within‐Day Jumping Performance and its Relationship With Strength Level.” International Journal of Sports Medicine 44, no. 1: 38–47. 10.1055/a-1898-4888. 35820447

[ejsc70286-bib-0012] Harrison, P. W. , L. P. James , D. G. Jenkins , M. R. McGuigan , R. W. Schuster , and V. G. Kelly . 2021. “Time Course of Neuromuscular, Hormonal, and Perceptual Responses Following Moderate‐ and High‐Load Resistance Priming Exercise.” International Journal of Sports Physiology and Performance 16, no. 10: 1472–1482. 10.1123/ijspp.2020-0646. 33761461

[ejsc70286-bib-0013] Harrison, P. W. , L. P. James , M. R. McGuigan , D. G. Jenkins , and V. G. Kelly . 2019. “Resistance Priming to Enhance Neuromuscular Performance in Sport: Evidence, Potential Mechanisms and Directions for Future Research.” Sports Medicine Springer International Publishing 49, no. 10: 1499–1514. 10.1007/s40279-019-01136-3. 31203499

[ejsc70286-bib-0014] Holmberg, P. M. , P. W. Harrison , D. G. Jenkins , and V. G. Kelly . 2023. “Factors Modulating the Priming Response to Resistance and Stretch‐Shortening Cycle Exercise Stimuli.” Strength and Conditioning Journal 45, no. 2: 188–206. 10.1519/ssc.0000000000000728.

[ejsc70286-bib-0015] Huang, T. Y. , F. T. Chen , R. H. Li , et al. 2022. “Effects of Acute Resistance Exercise on Executive Function: A Systematic Review of the Moderating Role of Intensity and Executive Function Domain.” Sports Medicine—Open Springer Science and Business Media Deutschland GmbH 8, no. 1: 141. 10.1186/s40798-022-00527-7. PMC9732176 36480075

[ejsc70286-bib-0016] Karlsen, R. H. , J. E. Karr , S. B. Saksvik , et al. 2022. “Examining 3‐Month Test‐Retest Reliability and Reliable Change Using the Cambridge Neuropsychological Test Automated Battery.” Applied Neuropsychology:Adult 29, no. 2: 146–154. 10.1080/23279095.2020.1722126. 32083946

[ejsc70286-bib-0019] Loturco, I. , P. Zmijewski , V. P. Mercer , et al. 2025. “Effects of Light‐Load vs. Heavy‐Load Jump Squats as Priming Activities in Olympic Female Rugby Sevens Players.” Biology of Sport. 10.5114/biolsport.2025.142645. PMC11963119 40182726

[ejsc70286-bib-0020] MacDonald, L. A. , and C. L. Minahan . 2016. “Indices of Cognitive Function Measured in Rugby Union Players Using a Computer‐Based Test Battery.” Journal of Sports Sciences 34, no. 17: 1669–1674. 10.1080/02640414.2015.1132003. 26756946

[ejsc70286-bib-0021] Mahoney, C. , H. Taylor , and R. Kanarek . 2005. “6 the Acute Effects of Meals on Cognitive Performance.” Nutritional Neuroscience: 73–91. https://psycnet.apa.org/doi/10.1201/9780203564554.ch6.

[ejsc70286-bib-0022] Mason, B. , A. McKune , K. Pumpa , and N. Ball . 2020. “The Use of Acute Exercise Interventions as Game Day Priming Strategies to Improve Physical Performance and Athlete Readiness in Team‐Sport Athletes: A Systematic Review.” Sports Medicine Springer Science and Business Media Deutschland GmbH 50: 1943–1962. 10.1007/s40279-020-01329-1. 32779102

[ejsc70286-bib-0023] Mason, B. R. J. , C. K. Argus , B. Norcott , and N. B. Ball . 2017. “Resistance Training Priming Activity Improves Upper‐Body Power Output in Rugby Players.” Journal of Strength and Conditioning Research 31, no. 4: 913–920. 10.1519/jsc.0000000000001552. 27386962

[ejsc70286-bib-0024] McLellan, T. M. , J. A. Caldwell , and H. R. Lieberman . 2016. “A Review of Caffeine’s Effects on Cognitive, Physical and Occupational Performance.” Neuroscience & Biobehavioral Reviews 71, no. 1: 294–312. 10.1016/j.neubiorev.2016.09.001. 27612937

[ejsc70286-bib-0025] Modric, T. , C. Carling , C. Lago‐Peñas , Š Versic , R. Morgans , and D. Sekulic . 2023. “To Train or Not to Train (On Match Day)? Influence of a Priming Session on Match Performance in Competitive Elite‐Level Soccer.” Journal of Sports Sciences 41, no. 18: 1726–1733. 10.1080/02640414.2023.2296741. 38115752

[ejsc70286-bib-0026] Nutt, F. , S. P. Hills , M. Russell , et al. 2022. “Morning Resistance Exercise and Cricket‐Specific Repeated Sprinting Each Improve Indices of Afternoon Physical and Cognitive Performance in Professional Male Cricketers.” Journal of Science and Medicine in Sport 25, no. 2: 162–166. 10.1016/j.jsams.2021.08.017. 34535402

[ejsc70286-bib-0027] Owen, N. , J. Watkins , L. Kilduff , H. Bevan , and M. Bennett . 2014. “Development of a Criterion Method to Determine Peak Mechanical Power Output in a Countermovement Jump.” Journal of Strength & Conditioning Research 28, no. 6: 1552–1558. 10.1519/jsc.0000000000000311. 24276298

[ejsc70286-bib-0028] Pereira, L. A. , P. Zmijewski , A. Golas , K. Kotuła , M. R. McGuigan , and I. Loturco . 2025. “Priming Exercises and Their Potential Impact on Speed and Power Performance: A Narrative Review.” Journal of Human Kinetics. 10.5114/jhk/204371. PMC12360937 40837515

[ejsc70286-bib-0029] Pullinger, S. A. , E. L. Brocklehurst , R. P. Iveson , et al. 2014. “Is There a Diurnal Variation in Repeated Sprint Ability on a Non‐Motorised Treadmill?” Chronobiology International 31, no. 3: 421–432. 10.3109/07420528.2013.865643. 24328815

[ejsc70286-bib-0030] Pullinger, S. A. , J. Oksa , L. F. Clark , et al. 2018. “Diurnal Variation in Repeated Sprint Performance Cannot be Offset When Rectal and Muscle Temperatures are at Optimal Levels (38.5°C).” Chronobiology International 35, no. 8: 1054–1065. 10.1080/07420528.2018.1454938. 29566344

[ejsc70286-bib-0031] Russell, M. , A. King , R. M. Bracken , C. J. Cook , T. Giroud , and L. P. Kilduff . 2016. “A Comparison of Different Modes of Morning Priming Exercise on Afternoon Performance.” International Journal of Sports Physiology and Performance 11, no. 6: 763–767. 10.1123/ijspp.2015-0508. 26658460

[ejsc70286-bib-0032] Scharfen, H. E. , and D. Memmert . 2019. “The Relationship Between Cognitive Functions and Sport‐Specific Motor Skills in Elite Youth Soccer Players.” Frontiers in Psychology 10, no. APR: 817. 10.3389/fpsyg.2019.00817. PMC6494938 31105611

[ejsc70286-bib-0033] Smith, P. J. , A. C. Need , E. T. Cirulli , O. Chiba‐Falek , and D. K. Attix . 2013. “A Comparison of the Cambridge Automated Neuropsychological Test Battery (CANTAB) With ‘Traditional’ Neuropsychological Testing Instruments.” Journal of Clinical and Experimental Neuropsychology 35, no. 3: 319–328. 10.1080/13803395.2013.771618. 23444947

[ejsc70286-bib-0034] Spring, B. , O. Maller , J. Wurtman , L. Digman , and L. Cozolino . 1982. “Effects of Protein and Carbohydrate Meals on Mood and Performance: Interactions With Sex and age.” Journal of Psychiatric Research 17, no. 2: 155–167. 10.1016/0022-3956(82)90017-6. 6764932

[ejsc70286-bib-0035] Stavropoulos, T. , Z. Nikolaos , K. Georgia‐Kassandra , et al. 2025. “Effects of Priming With Light vs. Heavy Loads on Weightlifting Performance.” Journal of Functional Morphology and Kinesiology 10, no. 1: 52. 10.3390/jfmk10010052. PMC11843885 39982292

[ejsc70286-bib-0036] Teo, W. , M. J. Newton , and M. R. McGuigan . 2011. “Circadian Rhythms in Exercise Performance: Implications for Hormonal and Muscular Adaptation.” Journal of Sports Science and Medicine 10, no. 4: 600–606. https://pubmed.ncbi.nlm.nih.gov/24149547/. PMC3761508 24149547

[ejsc70286-bib-0037] Torgersen, J. , H. Flaatten , B. A. Engelsen , and A. Gramstad . 2012. “Clinical Validation of Cambridge Neuropsychological Test Automated Battery in a Norwegian Epilepsy Population.” Journal of Behavioral and Brain Science 2, no. 1: 108–116. 10.4236/jbbs.2012.21013.

[ejsc70286-bib-0038] Tsuk, S. , Y. Netz , A. Dunsky , et al. 2019. “The Acute Effect of Exercise on Executive Function and Attention: Resistance Versus Aerobic Exercise.” Advances in Cognitive Psychology 15, no. 3: 208–215. 10.5709/acp-0269-7. PMC6776756 32161629

[ejsc70286-bib-0039] Villarreal, E. S. S. de , J. J. González‐Badillo , and M. Izquierdo . 2007. “Optimal Warm‐Up Stimuli of Muscle Activation to Enhance Short and Long‐Term Acute Jumping Performance.” European Journal of Applied Physiology 100, no. 4: 393–401. 10.1007/s00421-007-0440-9. 17394010

[ejsc70286-bib-0040] Walczak, K. , J. Krasnoborska , S. Samojedny , et al. 2024. “Effect of Creatine Supplementation on Cognitive Function and Mood.” Journal of Education, Health and Sport 73: 51712. 10.12775/jehs.2024.73.51712.

[ejsc70286-bib-0041] Waldron, M. , P. R. Worsfold , C. Twist , and K. Lamb . 2013. “The Relationship Between Physical Abilities, Ball‐Carrying and Tackling Among Elite Youth Rugby League Players.” Journal of Sports Sciences 32, no. 6: 542–549. 10.1080/02640414.2013.841975. 24070519

[ejsc70286-bib-0042] Wells, A. S. , N. W. Read , M. Fried , J. Borovicka , and M. D’Amato . 1997. “Effects of a Specific CCK‐A Antagonist, Loxiglumide, on Postprandial Mood and Sleepiness.” Journal of Psychopharmacology 11, no. 3: 241–246. 10.1177/026988119701100307. 9305416

